# Chemoimmunotherapy Outcomes and Prognostic Factors in Patients with Advanced, Low PD-L1–Expressing Non–Small Cell Lung Cancer

**DOI:** 10.1158/2767-9764.CRC-25-0157

**Published:** 2025-07-23

**Authors:** Tae Hata, Tadaaki Yamada, Yasuhiro Goto, Akihiko Amano, Yoshiki Negi, Satoshi Watanabe, Naoki Furuya, Tomohiro Oba, Tatsuki Ikoma, Akira Nakao, Keiko Tanimura, Hirokazu Taniguchi, Akihiro Yoshimura, Tomoya Fukui, Daiki Murata, Kyoichi Kaira, Shinsuke Shiotsu, Makoto Hibino, Asuka Okada, Yusuke Chihara, Hayato Kawachi, Takashi Kijima, Koichi Takayama

**Affiliations:** 1 Department of Pulmonary Medicine, Graduate School of Medical Science, Kyoto Prefectural University of Medicine, Kyoto, Japan.; 2 Department of Respiratory Medicine, Fujita Health University School of Medicine, Toyoake, Japan.; 3 Department of Respiratory Medicine, Kurashiki Central Hospital, Kurashiki, Japan.; 4 Department of Respiratory Medicine and Hematology, School of Medicine, Hyogo Medical University, Nishinomiya, Japan.; 5 Department of Respiratory Medicine and Infectious Diseases, Niigata University Graduate School of Medical and Dental Sciences, Niigata, Japan.; 6 Division of Respiratory Medicine, Department of Internal Medicine, St. Marianna University School of Medicine, Kawasaki, Japan.; 7 Department of Respiratory Medicine, Saitama Red Cross Hospital, Saitama, Japan.; 8 Department of Thoracic Oncology, Kansai Medical University, Hirakata, Japan.; 9 Department of Respiratory Medicine, Fukuoka University Hospital, Fukuoka, Japan.; 10 Department of Medical Oncology, Fukuchiyama City Hospital, Fukuchiyama, Japan.; 11 Department of Respiratory Medicine, Nagasaki University Graduate School of Biomedical Sciences, Nagasaki, Japan.; 12 Department of Respiratory Medicine, Japanese Red Cross Kyoto Daini Hospital, Kyoto, Japan.; 13 Department of Respiratory Medicine, Shonan Kamakura General Hospital, Kanagawa, Japan.; 14 Division of Respirology, Neurology, and Rheumatology, Department of Internal Medicine, Kurume University School of Medicine, Kurume, Japan.; 15 Department of Respiratory Medicine, International Medical Center, Saitama Medical University, Hidaka, Japan.; 16 Department of Respiratory Medicine, Japanese Red Cross Kyoto Daiichi Hospital, Kyoto, Japan.; 17 Department of Respiratory Medicine, Shonan Fujisawa Tokushukai Hospital, Kanagawa, Japan.; 18 Department of Respiratory Medicine, Saiseikai Suita Hospital, Suita, Japan.; 19 Department of Respiratory Medicine, Uji-Tokushukai Medical Center, Uji, Japan.

## Abstract

Chemoimmunotherapy is recommended for patients with non–small cell lung cancer (NSCLC) with low PD-L1 expression, but the effect of additional immunotherapy is heterogeneous in this population. To identify patients who do not benefit from the addition of immune checkpoint inhibitors (ICI) to chemotherapy, we conducted a retrospective study at 19 institutions in Japan. We analyzed 851 patients with advanced NSCLC with a PD-L1 tumor proportion score of 1% to 49% who received chemoimmunotherapy (*n* = 504) or chemotherapy (*n* = 347) between March 2017 and June 2022. After adjustment by propensity score matching, the median overall survival (OS) was 22.3 months in the chemoimmunotherapy group and 17.0 months in the chemotherapy-alone group (*P* = 0.01). Multivariate analysis showed that among 12 clinical factors, liver metastases (*P* = 0.001) and history of antibiotic use (*P* = 0.02) were independently associated with shorter OS in the chemoimmunotherapy group. Patients with liver metastases (OS, *P* = 0.4; progression-free survival, *P* = 0.06) or history of antimicrobial use (OS, *P* = 0.24; progression-free survival, *P* = 0.09) did not benefit from the addition of ICI to chemotherapy. Patients with a history of antimicrobial use experienced more severe pneumonitis with chemoimmunotherapy than all patients (*P* = 0.04). This cohort study showed that liver metastases and prior antimicrobial therapy are the most important clinical factors that attenuate the efficacy of chemoimmunotherapy in patients with low PD-L1 expression.

**Significance:**

This study shows that liver metastases and prior antibiotic use are key factors for chemoimmunotherapy in advanced NSCLC cases with low PD-L1 expression. They reduce the benefit of adding ICIs to chemotherapy, underscoring the need for new strategies to improve ICI efficacy in patients.

## Introduction

The clinical development of immune checkpoint inhibitors (ICI) has led to significant progress in cancer treatment. In the treatment of advanced non–small cell lung cancer (NSCLC), ICIs, alone or in combination with chemotherapy, have provided some patients with long-term survival not achieved with standard chemotherapy alone (1–7). In clinical practice, the expression of PD-L1 in tumor cells is widely used as a factor for predicting the efficacy of ICI in patients with NSCLC (8).

For patients with NSCLC and a PD-L1 tumor proportion score (TPS) of 1% to 49%, exhibiting low PD-L1 expression, sub-analyses of several clinical trials have reported that combination therapy involving chemotherapy and ICI prolongs progression-free survival (PFS) and overall survival (OS) as compared with chemotherapy or ICI monotherapy (7, 9–11). Therefore, the combination of chemotherapy and ICI is considered the first-line treatment for patients with low PD-L1 expression who are eligible for ICI administration.

Nevertheless, in a subset of patients with NSCLC with PD-L1 TPS of 1% to 49%, the effectiveness of ICI has been limited and concerns about side effects exist (12). A previous retrospective study in patients with NSCLC with PD-L1–negative or PD-L1–low expression reported that ICI combination chemotherapy significantly prolonged OS, whereas another report indicated poor efficacy of ICI combination chemotherapy in older patients with low PD-L1 expression (13, 14). Therefore, by identifying subgroups appropriately, conventional chemotherapy can be chosen as the first-line treatment, which is expected to achieve similar treatment effects, while avoiding toxicity. As a new predictive factor for efficacy, tumor-infiltrating lymphocytes have been reported to correlate significantly with the response to ICI, more than does tumor PD-L1 expression, in patients with NSCLC who have low PD-L1 expression. However, measuring these factors in routine clinical practice is challenging (15–17).

The aim of the study was to compare the efficacy and safety of ICI combination chemotherapy and platinum-based chemotherapy in patients with NSCLC with PD-L1 TPS between 1% and 49% in a real-world setting and to identify patients who do not require the addition of ICI to chemotherapy based on their clinical characteristics to advance personalized medicine in ICI therapy.

## Materials and Methods

### Patients and study design

We conducted a retrospective data collection from medical records at 19 participating institutions, including 10 university hospitals, in Japan. This study adhered to the Declaration of Helsinki and was conducted according to protocols approved by the Ethics Committee of Kyoto Prefectural University of Medicine for all centers by a collective central review (no. ERB-C-2934). Informed consent for the use of personal medical data was obtained through an opt-out method based on the disclosure document.

The inclusion criteria were (i) patients with stage IIIB, IIIC, IVA, IVB, or recurrent NSCLC according to the tumor–node–metastasis classification, eighth edition; (ii) patients who received either ICI combination platinum-doublet chemotherapy or platinum-doublet chemotherapy alone as first-line treatment between March 2017 and June 2022. Patients with *EGFR* mutations or anaplastic lymphoma kinase fusions were eligible after treatment with tyrosine kinase inhibitor; (iii) patients with a PD-L1 TPS of 1% to 49% as assessed using the 22C3 pharmDx antibody (clone 22C3; Dako); and (iv) patients with no prior administration of ICI. We excluded patients who received a combination of PD-1 and CTLA-4 antibodies only. The data cutoff date for follow-up was January 5, 2024.

### Assessment

The information on patient background, clinicopathologic characteristics, concomitant medication, and treatment history was extracted from the patient’s medical records. Information on concomitant medication consisted of the use of proton pump inhibitors (PPI) at the start of the treatment, systemic antimicrobial treatment within 1 month prior to treatment, and corticosteroids (prednisone equivalent to 10 mg daily for at least 24 hours) at the start of treatment (excluding premedication). The rationale for the 30-day cutoff point for a history of antimicrobial therapy before treatment initiation was based on longitudinal bacteriologic data and a systematic review of the prognostic value of immunotherapy (Supplementary Methods S1; refs. 18–20). Tumor response was evaluated every 6 to 9 weeks by clinicians at each institution, using CT, according to the RECIST version 1.1. OS was defined as from the date of treatment initiation up to death from any cause, and PFS was defined as from the date of treatment initiation up to clinical or radiological disease progression or death from any cause. Patients with no documented clinical or radiological disease progression or who were alive were censored at the last follow-up date. Adverse events (AE) were graded according to Common Terminology Criteria for Adverse Events version 5.0 by clinicians at each institution. We extracted all AEs above grade 3 and pneumonitis above grade 1.

### Statistical analysis

Categorical variables were compared using the Fisher exact test and continuous variables were compared using the Mann–Whitney U test. PFS and OS were evaluated using the Kaplan–Meier method, and the respective differences were determined by the log-rank test. HRs in univariate and multivariate analyses related to PFS and OS were evaluated with Cox proportional hazards regression models. Statistical significance was set as two-tailed *P* < 0.05.

We performed propensity score matching (PSM) to match measurable confounders between the two groups. We calculated a propensity score by logistic analysis using 15 variables (Supplementary Methods S1). A one-to-one match was then obtained using the nearest neighbor matching algorithm without replacement; the match was constrained to be within the caliper width of 0.2 times the SD of the log of the propensity score (21).

To identify patients who would not benefit from the addition of ICI to chemotherapy, univariate and multivariate analyses of both PFS and OS were performed to identify factors that significantly shortened survival in the ICI–chemotherapy group. Finally, PSM was performed again on those factors to compare survival between those receiving ICI–chemotherapy and those receiving chemotherapy alone.

All analyses were performed using SPSS version 27 (IBM SPSS Inc., RRID: SCR_002865) and GraphPad Prism version 10 (GraphPad Software, RRID: SCR_002798).

### Data availability

The datasets generated during and/or analyzed during the current study are available from the corresponding author on reasonable request.

## Results

### Patient characteristics

Of a total of 883 cases, 851 were included in the analysis (Supplementary Fig. S1). The median age was 70 years (range, 36–89 years), 639 (75%) were male, 786 (93%) had performance status 0 or 1, and 529 (62%) had adenocarcinoma. Of the patients, 504 (59%) received ICI–chemotherapy and 347 (41%) received chemotherapy alone (Supplementary Table S1). The characteristics of the ICI–chemotherapy and chemotherapy-alone groups before and after adjustment by PSM are shown in Table 1 and Supplementary Table S2. In the ICI–chemotherapy group, 335 patients (66%) received pembrolizumab, 99 (20%) received atezolizumab, and 69 (14%) received ipilimumab plus nivolumab (Supplementary Table S3). OS events (death) occurred in 283 patients in the ICI–chemotherapy group and 260 in the chemotherapy-alone group, and PFS events (disease progression or death) occurred in 374 patients in the ICI–chemotherapy group and 292 in the chemotherapy-alone group. The median follow-up duration was 46.7 (7.2–114.5) months for all patients.

**Table 1:** Patient characteristics

Characteristic	ICI plus chemotherapy*N* = 504*N* (%)	Chemotherapy*N* = 347*N* (%)	*P* value
Median age, years (range)	70 (36–89)	70 (39–88)	0.58
Sex	​	​	​
Female	118 (23)	94 (27)	0.23
Male	386 (77)	253 (73)	
ECOG performance status	​	​	​
0–1	468 (93)	318 (92)	0.51
2–4	36 (7)	29 (8)	
Smoking history	​	​	​
Never	72 (14)	66 (19)	0.07
Former/current	432 (86)	281 (81)	
Histology	​	​	​
Adenocarcinoma	322 (64)	207 (60)	0.54
Squamous	140 (28)	106 (31)	
NOS	19 (4)	18 (5)	
Others	23 (5)	16 (5)	
Disease stage	​	​	​
IIIB–IV	420 (83)	269 (78)	0.04
Recurrence	84 (17)	78 (22)	
EGFR mutation status	​	​	​
Positive	50 (10)	64 (18)	<0.001
Negative	401 (80)	231 (67)	
Unknown	53 (10)	52 (15)	
PD-L1 status	​	​	​
1%–24%	378 (75)	262 (76)	0.81
25%–49%	124 (25)	82 (24)	
Unknown among 1%–49%	2 (0.3)	3 (0.8)	
Interstitial pneumonia	​	​	​
Yes	12 (2)	67 (19)	<0.001
No	492 (98)	280 (81)	
Brain metastases	​	​	​
Yes	92 (18)	63 (18)	1.0
No	412 (82)	284 (82)	
Liver metastases	​	​	​
Yes	55 (11)	42 (12)	0.59
No	449 (89)	305 (88)	
PPI	​	​	​
Administered	165 (33)	130 (37)	0.16
Not administered	339 (67)	217 (63)	
ATBs	​	​	​
Administered	83 (16)	42 (12)	0.09
Not administered	421 (84)	305 (88)	
Steroids and/or immunosuppressant	​	​	​
Administered	34 (7)	37 (11)	0.04
Not administered	470 (93)	309 (89)	

Abbreviations: ECOG, Eastern Cooperative Oncology Group; NOS, not otherwise specified.

### Efficacy

**Figure 1:**
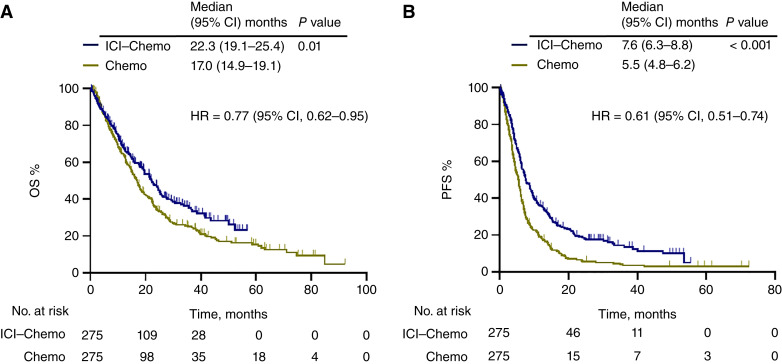
Treatment outcomes adjusted by PSM in all patients. The Kaplan–Meier curves provide estimates of OS (**A**) and PFS (**B**) of the 550 patients stratified by treatment with ICI plus chemotherapy (ICI–Chemo) and platinum-based chemotherapy (Chemo).

Analysis of 851 patients in the ICI–chemotherapy and chemotherapy-alone groups showed that the median OS [22.3; 95% confidence interval (CI), 19.6–25.0 months vs. 16.7; 95% CI, 15.1–18.4 months and HR = 0.75; 95% CI, 0.63–0.89; *P* < 0.001] and median PFS (7.5; 95% CI, 6.4–8.5 months vs. 5.6; 95% CI, 5.0–6.1 months and HR = 0.63; 95% CI, 0.54–0.73; *P* < 0.001) were both significantly longer in the ICI–chemotherapy group (Supplementary Fig. S2). PSM resulted in 550 matched cases, 275 in each group. Based on analysis of the matched groups, the objective response rates were 49% and 31% (*P* < 0.001), and disease control rates were 80% and 72% (*P* = 0.04) in the ICI–chemotherapy and chemotherapy-alone groups, respectively (Supplementary Table S4). Furthermore, even after adjustment for PSM, the ICI–chemotherapy group showed significantly prolonged median OS (22.3; 95% CI, 19.1–25.4 months vs. 17.0; 95% CI, 14.9–19.1 months and HR = 0.77; 95% CI, 0.62–0.95; *P* = 0.01) and median PFS (7.6; 95% CI, 6.3–8.8 months and 5.5; 95% CI, 4.8–6.2 months and HR = 0.61; 95% CI, 0.51–0.74; *P* < 0.001; Fig. 1).

### Poor prognostic factors among clinical background characteristics of patients undergoing combination therapy with ICI and chemotherapy

**Figure 2:**
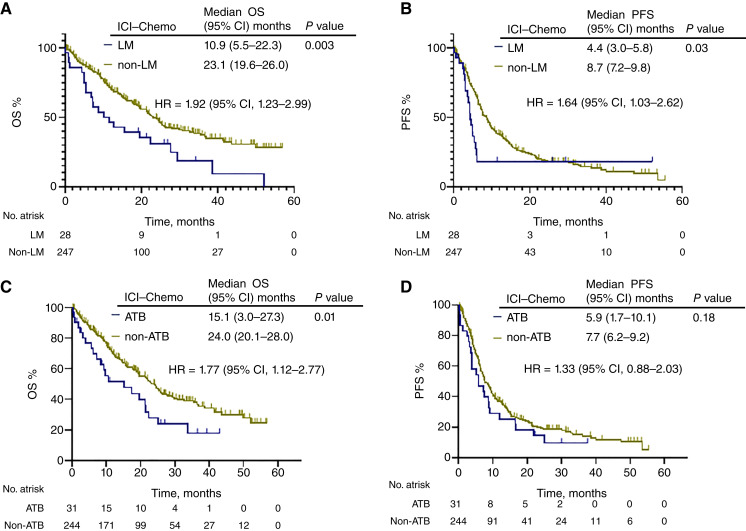
OS and PFS of patients with or without liver metastases or history of ATB therapy in the ICI plus chemotherapy group. The Kaplan–Meier curves provide estimates of OS (**A** and **C**) and PFS (**B** and **D**) of the ICI plus chemotherapy group, stratified by patients with or without liver metastases (**A** and **B**) or history of ATB (**C** and **D**). ICI–Chemo, ICI plus chemotherapy; LM, liver metastases.

We next analyzed the association between patient characteristics and OS and PFS in the ICI–chemotherapy group. The median OS was significantly shorter in squamous cell carcinoma (HR = 1.57; 95% CI, 1.13–2.20; *P* = 0.01), liver metastases (HR = 1.92; 95% CI, 1.23–2.99; *P* = 0.003), and history of antibiotics (ATB; HR = 1.77; 95% CI, 1.12–2.77; *P* = 0.01) in univariate analysis and significantly different in multivariate analysis for liver metastases (HR = 2.32; 95% CI, 1.39–3.87; *P* = 0.001) and history of ATB (HR = 1.92; 95% CI, 1.14–3.23; *P* = 0.01; Table 2). In patients treated with ICI chemotherapy, the group with liver metastases or prior antimicrobial use had a significantly shorter OS than the group without them (Fig. 2). The median PFS was significantly shorter in never smokers (HR = 1.45; 95% CI, 1.01–2.07; *P* = 0.04), *EGFR* mutation (HR = 1.65; 95% CI, 1.14–2.39; *P* = 0.04), and liver metastases (HR = 1.64; 95% CI, 1.03–2.62; *P* = 0.04) in univariate analysis, and multivariate analysis showed that *EGFR* mutation (HR = 1.66; 95% CI, 1.05–2.61; *P* = 0.03), performance status 0 to 1 (HR = 0.46; 95% CI, 0.25–0.86; *P* = 0.01), and liver metastases (HR = 2.47; 95% CI, 1.49–4.09; *P* < 0.001) were significantly different for median PFS (Table 3). In a multivariate analysis of OS and PFS in chemotherapy-treated patients, liver metastases and prior antimicrobial therapy were also poor prognostic factors (Supplementary Table S5).

**Table 2:** Univariate and multivariate analysis of OS in patients treated with ICI and chemotherapy

Characteristic	Patients, *N* (%)(*N* = 275)	Median OS (95% CI) months	Univariate analysis		Multivariate analysis	
			HR (95% CI)	*P* value	HR (95% CI)	*P* value
Age, years	​	​	​	​	​	​
<75	212	22.7 (18.9–26.5)	0.86 (0.59–1.27)	0.45	0.80 (0.54–1.32)	0.47
≥ 75	63	18.0 (10.8–25.3)	1 (reference)	NA	1 (reference)	NA
Sex	​	​	​	​	​	​
Female	67	21.6 (13.5–29.7)	1.01 (0.69–1.46)	0.98	1.28 (0.75–2.18)	0.36
Male	208	22.7 (18.9–26.5)	1 (reference)	NA	1 (reference)	NA
ECOG performance status	​	​	​	​	​	​
0–1	258	22.1 (18.9–25.3)	0.77 (0.39–1.51)	0.45	0.76 (0.36–1.63)	0.48
2–4	17	25.4 (0–58.2)	1 (reference)	NA	1 (reference)	NA
Smoking history	​	​	​	​	​	​
Never	43	25.8 (6.0–45.6)	0.77 (0.48–1.21)	0.25	0.64 (0.33–1.27)	0.2
Former/current	232	22.0 (18.2–25.8)	1 (reference)	NA	1 (reference)	NA
Histology	​	​	​	​	​	​
Squamous	75	17.7 (11.0–24.5)	1.57 (1.13–2.20)	0.01	1.36 (0.87–2.10)	0.17
Non-squamous	200	25.1 (20.8–29.4)	1 (reference)	NA	1 (reference)	NA
EGFR mutation status	​	​	​	​	​	​
Positive	43	22.1 (18.6–25.6)	1.08 (0.69–1.68)	0.75	1.14 (0.68–1.92)	0.62
Negative	198	24.4 (20.1–28.8)	1 (reference)	NA	1 (reference)	NA
Brain metastases	​	​	​	​	​	​
Yes	52	22.1 (18.4–25.8)	1.02 (0.69–1.52)	0.92	0.89 (0.55–1.44)	0.63
No	225	22.3 (16.0–28.7)	1 (reference)	NA	1 (reference)	NA
Liver metastases	​	​	​	​	​	​
Yes	28	10.9 (5.5–22.3)	1.92 (1.23–2.99)	0.003	2.32 (1.39–3.87)	0.001
No	247	23.1 (19.6–26.0)	1 (reference)	NA	1 (reference)	NA
Prior radiotherapy	​	​	​	​	​	​
Yes	47	21.6 (9.5–33.8)	1.19 (0.79–1.78)	0.41	1.22 (0.75–1.98)	0.42
No	228	22.3 (18.4–26.1)	1 (reference)	NA	1 (reference)	NA
PPI	​	​	​	​	​	​
Administered	91	25.1 (14.5–35.7)	1.04 (0.75–1.46)	0.81	0.97 (0.65–1.45)	0.88
Not administered	184	21.4 (18.7–24.2)	1 (reference)	NA	1 (reference)	NA
ATBs	​	​	​	​	​	​
Administered	31	15.1 (3.0–27.3)	1.77 (1.12–2.77)	0.01	1.92 (1.14–3.23)	0.01
Not administered	244	24.0 (20.1–28.0)	1 (reference)	NA	1 (reference)	NA
Steroids and/or immunosuppressant	​	​	​	​	​	​
Administered	23	23.1 (8.8–37.5)	0.92 (0.52–1.62)	0.77	0.97 (0.51–1.85)	0.93
Not administered	252	22.1 (18.7–25.5)	1 (reference)	NA	1 (reference)	NA

Abbreviations: ECOG, Eastern Cooperative Oncology Group; NA, not assessed.

**Table 3:** Univariate and multivariate analysis of PFS in patients treated with ICI and chemotherapy

Characteristic	Patients, *N* (%)(*N* = 275)	Median PFS (95% CI) months	Univariate analysis		Multivariate analysis	
			HR (95% CI)	*P* value	HR (95% CI)	*P* value
Age, years	​	​	​	​	​	​
<75	212	8.6 (7.2–10.0)	0.89 (0.64–1.23)	0.48	0.72 (0.50–1.04)	0.08
≥ 75	63	6.1 (5.5–6.7)	1 (reference)	NA	1 (reference)	NA
Sex	​	​	​	​	​	​
Female	67	6.4 (5.2–7.7)	1.30 (0.95–1.77)	0.10	0.94 (0.59–1.50)	0.79
Male	208	8.8 (7.1–10.6)	1 (reference)	NA	1 (reference)	NA
ECOG performance status	​	​	​	​	​	​
0–1	258	7.7 (6.2–9.2)	0.69 (0.39–1.20)	0.19	0.46 (0.25–0.86)	0.01
2–4	17	4.1 (0–9.4)	1 (reference)	NA	1 (reference)	NA
Smoking history	​	​	​	​	​	​
Never	43	7.0 (5.4–8.6)	1.45 (1.01–2.07)	0.04	1.17 (0.68–2.01)	0.57
Former/current	232	7.7 (6.0–9.4)	1 (reference)	NA	1 (reference)	NA
Histology	​	​	​	​	​	​
Squamous	75	7.1 (5.5–8.8)	1.17 (0.87–1.58)	0.31	1.13 (0.77–1.65)	0.53
Non-squamous	200	7.7 (6.1–9.3)	1 (reference)	NA	1 (reference)	NA
EGFR mutation status	​	​	​	​	​	​
Positive	43	6.5 (5.0–7.7)	1.65 (1.14–2.39)	0.01	1.66 (1.05–2.61)	0.03
Negative	198	8.7 (7.1–10.2)	1 (reference)	NA	1 (reference)	NA
Brain metastases	​	​	​	​	​	​
Yes	52	7.0 (4.7–9.3)	1.02 (0.72–1.46)	0.91	0.88 (0.58–1.34)	0.55
No	225	8.6 (7.2–10.1)	1 (reference)	NA	1 (reference)	NA
Liver metastases	​	​	​	​	​	​
Yes	28	4.4 (3.0–5.8)	1.64 (1.03–2.62)	0.04	2.47 (1.49–4.09)	0.0005
No	247	8.7 (7.2–9.8)	1 (reference)	NA	1 (reference)	NA
Prior radiotherapy	​	​	​	​	​	​
Yes	47	6.9 (5.3–8.5)	0.92 (0.64–1.34)	0.68	0.80 (0.52–1.24)	0.31
No	228	7.7 (6.5–8.9)	1 (reference)	NA	1 (reference)	NA
PPI	​	​	​	​	​	​
Administered	91	7.5 (6.2–8.7)	0.87 (0.64–1.16)	0.33	0.87 (0.62–1.22)	0.41
Not administered	184	8.0 (6.6–9.4)	1 (reference)	NA	1 (reference)	NA
ATBs	​	​	​	​	​	​
Administered	31	5.9 (1.7–10.0)	1.33 (0.88–2.03)	0.18	1.29 (0.80–2.07)	0.3
Not administered	244	7.7 (6.2–9.2)	1 (reference)	NA	1 (reference)	NA
Steroids and/or immunosuppressant	​	​	​	​	​	​
Administered	23	6.4 (3.1–9.7)	1.26 (0.80–2.01)	0.32	1.33 (0.78–2.29)	0.3
Not administered	252	7.7 (6.5–8.9)	1 (reference)	NA	1 (reference)	NA

Abbreviation: ECOG, Eastern Cooperative Oncology Group; NA, not assessed.

**Figure 3:**
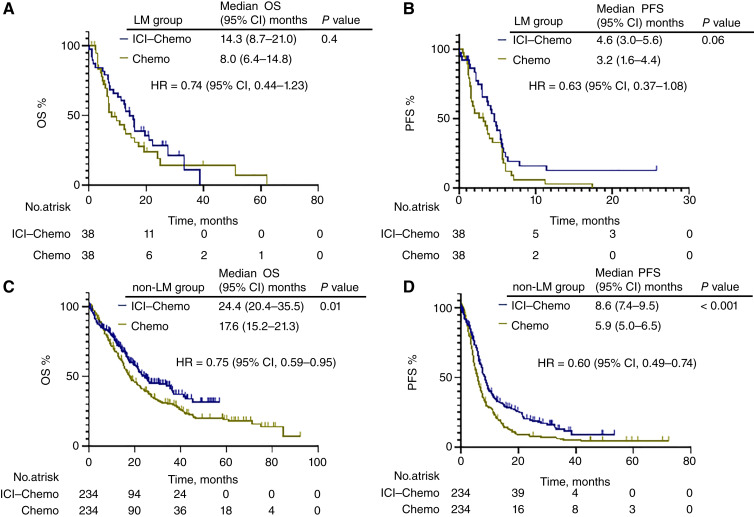
Treatment outcomes according to liver metastases. The Kaplan–Meier curves provide estimates of OS (**A**) and PFS (**B**) of the patients with liver metastases stratified by treatment with ICI plus chemotherapy and chemotherapy (*n* = 76). OS (**C**) and PFS (**D**) of the patients without liver metastases (non-LM) stratified by treatment with ICI plus chemotherapy and chemotherapy (*n* = 468). Chemo, chemotherapy; ICI–Chemo, ICI plus chemotherapy; LM, liver metastases.

**Figure 4:**
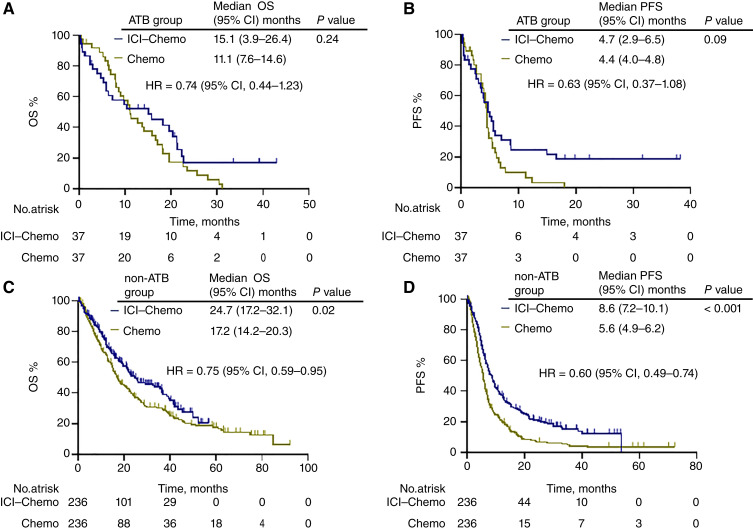
Treatment outcomes according to history of ATB therapy. The Kaplan–Meier curves provide estimates of OS (**A**) and PFS (**B**) of the patients with a history of ATB therapy stratified by treatment with ICI plus chemotherapy and chemotherapy (*n* = 74). OS (**C**) and PFS (**D**) of the patients without a history of ATB therapy stratified by treatment with ICI plus chemotherapy and chemotherapy (*n* = 472). Chemo, chemotherapy; ICI–Chemo, ICI plus chemotherapy.

Based on the results of the multivariate analysis of OS, the outcomes of the ICI–chemotherapy and chemotherapy-alone groups were compared, focusing on whether the patients had liver metastases and a history of ATB use. After PSM weighting, 38 patients with liver metastases (the liver metastasis group) and 234 patients without liver metastases (the non–liver metastasis group) were included in each group (Supplementary Table S6). Similarly, 37 patients with a history of ATB therapy (the ATB group) and 236 patients without a history of ATB therapy (the non-ATB group) were included in each group (Supplementary Table S7). As a result, there was no significant difference in the median OS [liver metastasis group: 14.3 (95% CI, 8.7–21.0) months vs. 8.0 (95% CI, 6.4–14.8) months; HR = 0.74; 95% CI, 0.44–1.23; *P* = 0.4 and ATB group: 15.1 (95% CI, 3.9–26.4) months vs. 11.1 (95% CI, 7.6–14.6) months; HR = 0.74; 95% CI, 0.44–1.23; *P* = 0.24] and median PFS [liver metastasis group: 4.6 (95% CI, 3.0–5.6) months vs. 3.2 (95% CI, 1.6–4.4) months; HR = 0.63; 95% CI, 0.37–1.08; *P* = 0.06 and ATB group: 4.7 (95% CI, 2.9–6.5) months vs. 4.4 (95% CI, 4.0–4.8) months; HR = 0.63; 95% CI, 0.37–1.08; *P* = 0.09] between the ICI–chemotherapy and chemotherapy groups in patients with liver metastases or a history of ATBs, respectively. In contrast, the ICI–chemotherapy group in patients with liver metastases or a history of ATBs showed significantly prolonged OS [non–liver metastasis group: 24.4 (95% CI, 20.4–35.5) months vs. 17.6 (95% CI, 15.2–21.3) months; HR = 0.75; 95% CI, 0.59–0.95; *P* = 0.01, non-ATB group: 24.7 (95% CI, 17.2–32.1) months vs. 17.2 (95% CI, 14.2–20.3) months; HR = 0.75; 95% CI, 0.59–0.95; *P* = 0.02] and PFS [non–liver metastasis group: 8.6 (95% CI, 7.4–9.5) months vs. 5.9 (95% CI, 5.0–6.5) months; HR = 0.60; 95% CI, 0.49–0.74; *P* < 0.001, non-ATB group: 8.6 (95% CI, 7.2–10.1) months vs. 5.6 (95% CI, 4.9–6.2) months; HR = 0.60; 95% CI, 0.49–0.74; *P* < 0.001], respectively (Figs. 3 and 4).

In patients with liver metastases or a history of ATB use, brain metastases were more frequent in the ICI–chemotherapy group than in the chemotherapy group, but these differences were not statistically significant (liver metastases: *P* = 0.32; history of ATB use: *P* = 0.37). Among patients with liver metastases who had previously received brain radiotherapy, four cases (11%) were observed in the ICI–chemotherapy group and one case (3%) in the chemotherapy group (*P* = 0.36). Among patients with a history of ATB use, brain radiotherapy was administered prior to treatment in three cases (8%) in the ICI–chemotherapy group and two cases (5%) in the chemotherapy group (*P* = 0.48).

### Details of antimicrobial administration history

The most common type of antimicrobial was β-lactam ATBs in 86 cases (68%), followed by quinolone ATBs in 18 cases (14%; Supplementary Table S8). Pneumonia was the most common reason for antimicrobial administration (61 cases, 48%), followed by prophylactic administration for biopsy (40 cases, 32%). The primary treatment modalities were hospitalization in 71 cases (56%), outpatient visits in 39 cases (31%), and unknown in 16 cases (13%). The median duration of antimicrobial therapy was 7 days (range, 1–584 days). Most patients were on antimicrobials for short periods of time and were not severely ill. These results suggest that antimicrobial therapy may reduce the efficacy of ICI for prophylactic administration, mild infections, and even short-term administration.

### Safety

In the overall population, the incidence of grade 3 or higher AEs was around 40% in both the ICI–chemotherapy and chemotherapy-alone groups but was more than 50% in the ATB group, with more AEs reported in the ATB group (Table 4; all patients, 38% vs. ATB group, 55%; *P* = 0.005). No increase in AEs was observed in the non–liver metastasis group (33%). Comparing the overall group with the ATB group, the incidence of anemia and lung infection of grade 3 or higher was significantly higher in the ATB group (anemia, 19% vs. 5%, *P* < 0.001; lung infection, 11% vs. 2%, *P* = 0.001). In addition, the incidence of severe pneumonitis of grade 3 or higher was significantly higher in the ATB group than in the overall group when treated with ICI–chemotherapy (16% vs. 6%; *P* = 0.04; Supplementary Fig. S3; Supplementary Table S9). In all patients, the ICI–chemotherapy group had a higher discontinuation rate than the chemotherapy-alone group. A similar trend was observed in the analysis of the liver metastasis and ATB groups (overall: 26% vs. 14%; *P* < 0.001; liver metastases: 18% vs. 8%; *P* = 0.31; ATB: 24% vs. 11%; *P* = 0.22; Supplementary Table S10).

**Table 4:** Grade 3 or higher AEs in the population adjusted by PSM

AE	Patients *N* = 550		ATB group *N* = 74		LM group *N* = 76	
	ICI plus chemotherapy*N* = 275*n* (%)	Chemotherapy*N* = 275*n* (%)	ICI plus chemotherapy*N* = 37*n* (%)	Chemotherapy*N* = 37*n* (%)	ICI plus chemotherapy*N* = 38*n* (%)	Chemotherapy*N* = 38*n* (%)
All	111 (40)	99 (36)	20 (54)	21 (57)	13 (34)	12 (32)
Hematologic AEs, *n* (%)	​	​	​	​	​	​
Neutropenia	44 (16)	48 (17)	9 (24)	8 (22)	5 (13)	6 (16)
Anemia	15 (5)	15 (5)	7 (19)	7 (19)	1 (3)	1 (3)
Thrombocytopenia	19 (7)	16 (6)	1 (3)	3 (8)	2 (5)	2 (5)
Non-hematologic AEs, *n* (%)	​	​	​	​	​	​
Febrile neutropenia	11 (4)	13 (5)	0 (0)	3 (8)	1 (3)	1 (3)
Skin disorder	10 (4)	3 (1)	1 (3)	1 (3)	1 (3)	0 (0)
Endocrine disorder	12 (4)	0 (0)	0 (0)	0 (0)	1 (3)	0 (0)
Liver dysfunction	8 (3)	7 (3)	1 (3)	0 (0)	2 (5)	0 (0)
Interstitial pneumonitis	17 (3)	15 (1)	6 (16)	1 (3)	0 (0)	1 (3)
Lung infection	5 (2)	7 (3)	4 (11)	4 (11)	0 (0)	1 (3)
Renal dysfunction	4 (1)	1 (0.3)	1 (3)	1 (3)	0 (0)	1 (3)
Electrolyte disturbance	1 (0.3)	4 (1)	1 (3)	2 (5)	1 (3)	1 (3)
Colitis	4 (1)	0 (0)	1 (3)	0 (0)	0 (0)	0 (0)
Anorexia	3 (1)	6 (2)	1 (3)	2 (5)	0 (0)	0 (0)
Nervous system disorder	3 (1)	2 (1)	0 (0)	1 (3)	0 (0)	0 (0)

## Discussion

In this multicenter study, we observed that ICI–chemotherapy is preferable to platinum-based chemotherapy in most patients with advanced NSCLC with PD-L1 TPS between 1% and 49% and from diverse characteristics. In addition, the key finding of this study is that no clear benefit of adding ICI to chemotherapy was observed in patients with liver metastases or those who received ATB within 1 month prior to treatment. It was also found that the incidence of severe interstitial pneumonitis increased when chemoimmunotherapy was given to patients with a history of antimicrobial use. To our knowledge, there have been no previous large-scale retrospective studies investigating the efficacy and prognostic predictors of ICI plus chemotherapy versus platinum-based treatment in patients with NSCLC and a PD-L1 TPS of 1% to 49%.

In a subanalysis of patients with PD-L1 TPS 1% to 49% in previous randomized trials, compared with chemotherapy, CheckMate 9LA (NCT03215706) resulted in a median OS of 15.8 vs. 11.0 months (HR = 0.73; 95% CI, 0.62–0.85) and KEYNOTE-189 (NCT02578680) resulted in a median OS of 21.8 vs. 12.1 months (HR = 0.65; 95% CI, 0.46–0.90; refs. 4, 7). First-line chemotherapy with atezolizumab for non–squamous cell carcinoma significantly prolonged median PFS in tumor cells 1/2 or tumor-infiltrating immune cells 1/2 as compared with chemotherapy (8.3 vs. 6.6 months; HR = 0.56; 95% CI, 0.41–0.77; ref. 11). Although these reflect the results of subanalyses, the additive effect of ICIs has been demonstrated consistently in various clinical trials in patients with low PD-L1 expression.

Several studies have reported that patients with NSCLC with liver metastases have a diminished efficacy of immunotherapy compared with patients without liver metastases (22–24). In a study examining the microtumorenvironment in patients with liver metastases as a possible cause, liver metastases were associated with decreased CD8-positive cell density at the invasive tumor margin (24). Meanwhile, a subgroup analysis of the IMpower150 trial and a meta-analysis of immunochemotherapy reported that patients with liver metastases experienced OS prolongation with ICI treatment (25, 26). Our study in patients with low PD-L1 expression suggests that the addition of ICI to chemotherapy should be carefully considered.

The effect of concomitant medications on the efficacy of immunotherapy alone has been reported with ATB, PPIs, and probiotic therapy (19, 20, 27–30). These drugs cause changes in the gut microbiota, and the gut microbiota, besides influencing the etiology of cancer, also plays an important role in the efficacy of therapy (31). Antimicrobial agents affect the composition of up to 30% of the bacterial species of the gut microbiota, causing marked changes in host and microbial ecological diversity that last for 2 years (32, 33). It has been demonstrated that the gut microbiota is disrupted in patients who receive ATB immediately before or after the initiation of immunotherapy, reducing the efficacy of antibodies against CTLA-4 and PD-1/PD-L1 (19, 20, 27). Several retrospective studies have reported significantly shorter OS with ICI in patients with a history of ATB (34, 35). In an analysis including multiple carcinomas, including NSCLC, it has also been reported that a history of prior ATBs, but not concomitant ICI and antimicrobial therapy, was associated with worse OS with ICI (36). Furthermore, previous reports indicate that patients receiving immunotherapy and chemotherapy have a higher incidence of immune-related AEs and AEs like multiorgan damage (37–39). Clinicians should optimize the use of antimicrobials for pretreatment patients with advanced cancer, as patients with lung cancer are more likely to receive antimicrobials than other carcinomas according to a large database study (40). Additionally, a previous study demonstrated that patients with a history of antimicrobial exposure who received probiotics experienced significantly longer OS and PFS compared with those who did not receive probiotics (29). In patients for whom antimicrobial therapy is unavoidable, the concomitant use of probiotics may be a potential strategy to prevent the reduction in the efficacy of ICIs and to maximize their therapeutic benefit.

Several previous studies have reported that ICI monotherapy is less effective in nonsmokers and extends the median OS more in smokers than in nonsmokers (41, 42). With regard to chemoimmunotherapy, some studies have shown efficacy in both smokers and nonsmokers, whereas others have reported that smoking quantity affects the efficacy of chemoimmunotherapy (43, 44). The results suggest that patients with low PD-L1 expression who have never smoked may benefit from chemoimmunotherapy as much as smokers.

Our study included patients with *EGFR* mutations, for whom ICI has been reported to have limited efficacy (45). Several studies reported that the efficacy of atezolizumab-bevacizumab-calboplatin-paclitaxel therapy versus chemotherapy alone in patients with *EGFR* mutations depends on PD-L1 expression (45–47). Although our study found no difference in prognosis between ICI–chemotherapy in patients with or without *EGFR* mutations, the addition of ICIs to chemotherapy for patients with *EGFR* mutations remains controversial.

In our study, PPI use was not associated with poor prognosis in patients treated with ICI–chemotherapy. Although several studies have reported that PPIs reduce the efficacy of ICI monotherapy, the relationship between ICI–chemotherapy and PPIs remains unclear (34, 48). In a study comparing ICI monotherapy with ICI–chemotherapy in patients with a PD-L1 TPS of 50% or more, PPI use was not an independent factor for PFS in patients receiving ICI–chemotherapy; the median OS and PFS were similar in the ICI–chemotherapy group with and without PPIs (28). These findings suggest that the concomitant use of PPIs may not significantly affect the efficacy of ICI combined with chemotherapy compared with ICI monotherapy.

The present study had several limitations. First, this was a retrospective study and unanticipated biases could have influenced the results. However, we took care to match covariates as closely as possible using PSM. Second, although the method of determining response was the same in all centers, based on RECIST 1.1, the timing of imaging to determine treatment response was not specified in advance. Third, the number of patients in this study with prior antimicrobial therapy and liver metastases was very small, 74 and 76 (after adjustment by PSM), respectively. This number limits statistical power, and further research is needed. Fourth, although approximately 80% of patients with a history of antimicrobial therapy had a performance status of 0 or 1 in both the ICI–chemotherapy and chemotherapy groups, the fact that some required antimicrobial treatment suggests they may have been in poorer general condition at the initiation of anticancer therapy, which could have affected their survival. Fifth, the increased incidence of pneumonitis associated with antimicrobial use cannot rule out the potential influence of confounding factors that can cause lung injury, such as pneumonia as the indication for antimicrobial therapy, preexisting interstitial lung abnormalities, or a history of thoracic radiotherapy.

Our results suggest that the addition of ICI to chemotherapy does not improve OS or PFS in patients with advanced NSCLC and a PD-L1 TPS of 1% to 49% who have liver metastases or have received prior antimicrobial therapy. Additionally, antimicrobial agents increased AEs. These factors are clinically significant for patients undergoing chemotherapy with ICI, and further research is necessary to optimize the benefits of ICI for this patient population.

### Glossary of terms

PD-L1 expression:When stained with 22C3 antibody, >50% are classified as high, <1% as negative, and 1% to 49% as low expression.

TC1/2 or IC1/2:It shows low expression of PD-L1 by SP142 antibody, a finding similar to 1% to 49% in 22C3 antibody.

## Supplementary Material

Supplementary Methods Supplementary Materials and Methods

Supplementary Table S1 Patients Characteristics

Supplementary Table S2 Patients Characteristics adjusted by Propensity Score Matching

Supplementary Table S3 Details of the treatment regimens

Supplementary Table S4 Effectiveness of treatments in the population adjusted by propensity score matching

Supplementary Table S5 Univariate and multivariate analysis of Overall Survival and Progression-Free Survival in patients treated with Chemotherapy

Supplementary Table S6 Characteristics in the Liver and non-Liver metastases Groups adjusted by propensity score matching

Supplementary Table S7 Characteristics in the Antibiotics and non-Antibiotics Groups adjusted by propensity score matching

Supplementary Table S8 Details of the antimicrobial administration history

Supplementary Table S9 Incidence of interstitial pneumonitis as an adverse event in the population adjusted by propensity score matching

Supplementary Table S10 Discontinuation due to adverse events and ECOG Performance status deterioration due to treatment in the population adjusted by propensity score matching

Supplementary Figure S1 Study Flow Diagram

Supplementary Figure S2 Overall survival and Progression-free survival of 851 patients according to treatments

Supplementary Figure S3 Incidence of Interstitial Pneumonitis as an adverse event
