# Supplementary material for: Chemoimmunotherapy Outcomes and Prognostic Factors in Patients with Advanced, Low PD-L1–Expressing Non–Small Cell Lung Cancer
Source: Cancer Res Commun. 2025 Jul 23;5(7):1203–14. doi: 10.1158/2767-9764.CRC-25-0157 (PMC12284348; doi:10.1158/2767-9764.CRC-25-0157)
Supplement: Supplementary Methods — Supplementary Materials and Methods [file crc-25-0157_supplementary_methods_suppsmm.docx]

**Supplementary Materials and Methods**

**Data Collection and Patients**

The following 19 institutions participated; Kyoto Prefectural University of Medicine, Fujita Medical College Hospital, Kurashiki Central Hospital, Uji Tokushukai Hospital, Osaka Saiseikai Suita Hospital, Kansai Medical University Hospital, Kyoto First Red Cross Hospital, Kyoto Second Red Cross Hospital, Kurume University Hospital, Saitama Medical University International Medical Center, Saitama Red Cross Hospital, Shonan Kamakura General Hospital, Shonan Fujisawa Tokushukai Hospital, Fukuchiyama Municipal Hospital, St. Marianna Medical University Hospital, Nagasaki University Hospital, Niigata University Hospital, Hyogo University Hospital, Fukuoka University Hospital.

The following information was obtained from the patient's medical records; sex, age, smoking history, histology, Eastern Cooperative Oncology Group performance status, type of oncogenic mutation, PD-L1 expression, medical history, presence of interstitial pneumonia, presence of malignancy within 5 years, presence of uncontrolled pleural or pericardial effusion, height, weight, location of distant metastases, history of radiation therapy, blood data, history of antibiotics (ATB) within 1 month prior to the start of treatment, concomitant of proton pump inhibitors, steroids or immunosuppressive drugs, regimen, treatment effect, dates of treatment initiation and discontinuation, details of secondary therapy, date of death, grade 1 or higher interstitial lung disease, grade 3 or higher adverse events.

Non-small cell lung cancer patients harboring genetic alterations were included in this study. Detection of EGFR mutations and ALK fusion genes was performed using the Oncomine Dx Target Test (Thermo Fisher Scientific, Waltham, MA, USA), the AmoyDx Pan Lung Cancer PCR Panel, or LC-SCRUM (UMIN000010234), a screening test for lung cancer conducted by the National Cancer Center. Consent forms are signed by all participating patients prior to LC-SCRUM testing. In addition, EGFR mutations were detected using the Cobas EGFR Mutation Test v2 (Roche Molecular Systems) and the Vysis ALK Break Apart FISH Probe Kit (Abbott Molecular) was used to detect ALK fusion genes.

**Treatments**

All regimens that included ICI were either pembrolizumab, atezolizumab, or nivolumab and ipilimumab in combination with platinum-doublet chemotherapy. Pembrolizumab and atezolizumab were administered intravenously at a dose of 200mg or 1500mg every 3 weeks with chemotherapy, respectively. Nivolumab was administered at 360 mg every 3 weeks and ipilimumab at 1 mg/kg every 6 weeks along with anticancer agents, and after 3 cycles, nivolumab and ipilimumab alone were continued.

**Criteria for a history of antimicrobial therapy prior to treatment initiation**

The cutoff point for a history of antimicrobial therapy prior to treatment initiation was set at 30 days for two reasons. First, longitudinal bacteriological data show that bacterial concentration and gut microbiota diversity tend to recover within four weeks after antimicrobial administration.

Second, evidence shows that, in cancer patients who received antimicrobials 30–60 days before ICI administration, a medication history within 30 days is associated with poor overall survival (OS) and progression-free survival (PFS). Systematic reviews examining the prognostic value of antimicrobials and immunotherapy, including the above studies, have examined antimicrobial exposure and time to initiation of therapy and found that antimicrobial exposure 30 days prior to initiation of therapy had the greatest impact on efficacy.

**Statistical analysis**

We performed propensity score matching (PSM) to match measurable confounders of patients between the ICI plus chemotherapy and chemotherapy groups. In all 851 patients and 729 patients with no history of ATB, the following 15 covariates were used to calculate propensity scores: age, Eastern Cooperative Oncology Group performance status, sex, smoking history, histology, stage (IIIB, IIIC, IVA, IVB or recurrence), presence or absence of brain metastases, EGFR mutation status, medication history (proton pump inhibitors, antimicrobials, steroids and immunosuppressants), history of interstitial pneumonia, history of autoimmune disease, history of malignancy within 5 years (except for curable disease), and irradiation history. In 754 patients with liver metastases, the following 12 covariates were used to calculate propensity scores: age, Eastern Cooperative Oncology Group performance status, sex, smoking history, histology, stage (IIIB, IIIC, IVA, IVB or recurrence), presence or absence of brain metastases, EGFR mutation status, medication history (proton pump inhibitors, antimicrobials, steroids and immunosuppressants),and irradiation history. In 97 patients with liver metastases, the following four covariates were used to calculate propensity scores: age, Eastern Cooperative Oncology Group performance status, sex and histology. In 126 patients with a history of antimicrobial therapy, the following six covariates were used to calculate propensity scores: age, Eastern Cooperative Oncology Group performance status, sex, smoking history, histology, and EGFR mutation status. A one-to-one match was then obtained using the nearest neighbor matching algorithm without replacement; the match was constrained to be within the caliper width of 0.2 times the standard deviation of the log of the propensity score. Covariate balance after matching was verified by calculating the standardized difference between the two groups.

To identify patients who did not require the addition of ICI to chemotherapy, univariate and multivariate analyses of both PFS and OS were performed to identify factors that significantly shortened survival in the ICI plus chemotherapy group. Finally, PSM was performed again based on all 851 cases for the factor that significantly shortened survival (prior antibiotic therapy), and survival was compared between the ICI plus chemotherapy and chemotherapy groups.
