# Supplementary material for: Chemoimmunotherapy Outcomes and Prognostic Factors in Patients with Advanced, Low PD-L1–Expressing Non–Small Cell Lung Cancer
Source: Cancer Res Commun. 2025 Jul 23;5(7):1203–14. doi: 10.1158/2767-9764.CRC-25-0157 (PMC12284348; doi:10.1158/2767-9764.CRC-25-0157)
Supplement: Supplementary Table S1 — Patients Characteristics [file crc-25-0157_supplementary_table_s1_suppst1.docx]

**Supplementary Table S1. Patients Characteristics**

| **Characteristic** | **Patients, No. (%) (N = 851)** |
| --- | --- |
| Median age [range], years | 70 [36–89] |
| Sex |  |
| Female | 212 (25) |
| Male | 639 (75) |
| ECOG performance status |  |
| 0 | 277 (33) |
| 1 | 509 (60) |
| 2 | 57 (7) |
| 3–4 | 8 (0.9) |
| Smoking history |  |
| Never | 138 (16) |
| Former/current | 713 (84) |
| Histology |  |
| Adenocarcinoma | 529 (62) |
| Squamous | 246 (29) |
| NOS | 38 (4) |
| Others | 38 (4) |
| Disease stage |  |
| IIIB, IIIC | 55 (6) |
| IVA, IVB | 634 (75) |
| Recurrence | 162 (19) |
| Oncogenic alterations |  |
| EGFR | 114 (13) |
| KRAS | 37 (4) |
| ALK | 9 (1) |
| Others or negative | 586 (69) |
| Unknown | 105 (12) |
| Brain metastases |  |
| Yes | 155 (18) |
| No | 696 (82) |
| Liver metastases |  |
| Yes | 97 (11) |
| No | 754 (89) |
| Treatment |  |
| ICI plus chemotherapy | 504 (59) |
| Chemotherapy | 347 (41) |

Abbreviations: ECOG, Eastern Cooperative Oncology Group; EGFR, Epidermal growth factor receptor; KRAS, Kirstenrat sarcoma viral oncogene homolog; ALK, anaplastic lymphoma kinase; ICI, Immune checkpoint inhibitor
