# Supplementary material for: Chemoimmunotherapy Outcomes and Prognostic Factors in Patients with Advanced, Low PD-L1–Expressing Non–Small Cell Lung Cancer
Source: Cancer Res Commun. 2025 Jul 23;5(7):1203–14. doi: 10.1158/2767-9764.CRC-25-0157 (PMC12284348; doi:10.1158/2767-9764.CRC-25-0157)
Supplement: Supplementary Table S2 — Patients Characteristics adjusted by Propensity Score Matching [file crc-25-0157_supplementary_table_s2_suppst2.docx]

**Supplementary Table S2. Patients Characteristics adjusted by Propensity Score Matching**

| **Characteristic** | **ICI plus Chemotherapy**  **N = 275**  **No. (%)** | **Chemotherapy**  **N = 275**  **No. (%)** | ***P* Value** |
| --- | --- | --- | --- |
| Median age (range) | 70 [36-89] | 69 [39-88] | 0.57 |
| Sex |  |  |  |
| Female | 67 (24) | 78 (28) | 0.33 |
| Male | 208 (76) | 197 (72) |  |
| ECOG performance status |  |  |  |
| 0–1 | 258 (94) | 256 (93) | 0.86 |
| 2–4 | 17 (6) | 19 (7) |  |
| Smoking history |  |  |  |
| Never | 43 (16) | 49 (18) | 0.57 |
| Former/current | 232 (84) | 226 (82) |  |
| Histology |  |  |  |
| Adenocarcinoma | 171 (62) | 175 (64) | 0.98 |
| Squamous | 75 (27) | 74 (27) |  |
| NOS | 20 (7) | 18 (7) |  |
| Others | 9 (3) | 8 (3) |  |
| Disease stage |  |  |  |
| IIIB– IV | 219 (80) | 225 (82) | 0.59 |
| Recurrence | 56 (20) | 50 (18) |  |
| EGFR mutation status |  |  |  |
| Positive | 43 (16) | 51 (19) | 0.57 |
| Negative | 198 (72) | 187 (68) |  |
| Unknown | 34 (12) | 37 (13) |  |
| PD-L1 status |  |  |  |
| 1–24% | 209 (76) | 210 (76) | 1.0 |
| 25–49% | 64 (23) | 63 (23) |  |
| Interstitial pneumonia |  |  |  |
| Yes | 12 (4) | 13 (5) | 1.0 |
| No | 263 (96) | 262 (95) |  |
| Brain metastases |  |  |  |
| Yes | 52 (19) | 53 (19) | 1.0 |
| No | 223 (81) | 222 (81) |  |
| Liver metastases |  |  |  |
| Yes | 28 (10) | 35 (13) | 0.42 |
| No | 247 (90) | 240 (87) |  |
| Proton pump inhibitor |  |  |  |
| Administered | 91 (33) | 105 (38) | 0.25 |
| Not administered | 184 (67) | 170 (62) |  |
| Antibiotics |  |  |  |
| Administered | 31 (11) | 34 (12) | 0.79 |
| Not administered | 244 (89) | 241 (88) |  |
| Steroids and/or immunosuppressant |  |  |  |
| Administered | 23 (8) | 27 (10) | 0.66 |
| Not administered | 252 (92) | 248 (90) |  |

Abbreviations: ECOG, Eastern Cooperative Oncology Group; ICI, Immune checkpoint inhibitor; NOS, not otherwise specified; EGFR, Epidermal growth factor receptor; PD-L1, programmed cell death ligand
