# Supplementary material for: Chemoimmunotherapy Outcomes and Prognostic Factors in Patients with Advanced, Low PD-L1–Expressing Non–Small Cell Lung Cancer
Source: Cancer Res Commun. 2025 Jul 23;5(7):1203–14. doi: 10.1158/2767-9764.CRC-25-0157 (PMC12284348; doi:10.1158/2767-9764.CRC-25-0157)
Supplement: Supplementary Table S3 — Details of the treatment regimens [file crc-25-0157_supplementary_table_s3_suppst3.docx]

**Supplementary Table S3.** **Details of the treatment regimens**

| **Treatment** | **All patients, N = 851**  **No. (%)** |
| --- | --- |
| **Immunotherapy plus chemotherapy** | 504 (59) |
| Pembrolizumab / Carboplatin / Paclitaxel or nab-Paclitaxel | 136 (16) |
| Pembrolizumab / Carboplatin / Pemetrexed | 148 (17) |
| Pembrolizumab / Cisplatin / Pemetrexed | 51 (6) |
| Atezolizumab /Carboplatin / Paclitaxel or nab-Paclitaxel | 22 (3) |
| Atezolizumab / Carboplatin / Paclitaxel or nab-Paclitaxel / Bevacizumab | 67 (8) |
| Atezolizumab / Carboplatin / Pemetrexed | 8 (1) |
| Atezolizumab / Carboplatin / Pemetrexed/ Bevacizumab | 1 (0.1) |
| Atezolizumab / Cisplatin / Pemetrexed | 1 (0.1) |
| Nivolumab / Ipilimumab / Carboplatin / Paclitaxel | 28 (3) |
| Nivolumab / Ipilimumab / Carboplatin /Pemetrexed | 37 (4) |
| Nivolumab / Ipilimumab / Cisplatin / Pemetrexed | 4 (0.4) |
| Nivolumab / Carboplatin / Paclitaxel / Bevacizumab | 1 (0.1) |
| **Chemotherapy** | 347 (41) |
| Carboplatin / Paclitaxel or nab-Paclitaxel | 136 (16) |
| Carboplatin / Paclitaxel or nab-Paclitaxel / Bevacizumab | 7 (1) |
| Carboplatin / Pemetrexed | 75 (9) |
| Carboplatin / Pemetrexed / Bevacizumab | 21 (2) |
| Carboplatin / S-1 | 21 (2) |
| Carboplatin / Gemcitabine or Etoposide or Irinotecan | 4 (0.5) |
| Cisplatin / Pemetrexed | 44^†^ (5) |
| Cisplatin / Pemetrexed / Bevacizumab | 27^††^ (3) |
| Cisplatin / S-1 | 6 (1) |
| Cisplatin / Docetaxel or Gemcitabine | 3 (0.3) |
| Cisplatin / Gemcitabine / Necitumumab | 3 (0.3) |

†　2 cases, †† 1 case; Drug change from Cisplatin to Carboplatin due to renal dysfunction
