# Supplementary material for: Chemoimmunotherapy Outcomes and Prognostic Factors in Patients with Advanced, Low PD-L1–Expressing Non–Small Cell Lung Cancer
Source: Cancer Res Commun. 2025 Jul 23;5(7):1203–14. doi: 10.1158/2767-9764.CRC-25-0157 (PMC12284348; doi:10.1158/2767-9764.CRC-25-0157)
Supplement: Supplementary Table S4 — Effectiveness of treatments in the population adjusted by propensity score matching [file crc-25-0157_supplementary_table_s4_suppst4.docx]

**Supplementary Table S4.** **Effectiveness of treatments in the population adjusted by propensity score matching**

|  | **ICI plus Chemotherapy**  **N = 275**  **No. (%)** | **Chemotherapy**  **N = 275**  **No. (%)** | ***P* Value** |
| --- | --- | --- | --- |
| Treatment response |  |  |  |
| Complete response | 6 (2) | 3 (1) | 0.5 |
| Partial response | 128 (47) | 83 (30) | < 0.001 |
| Stable disease | 59 (21) | 88 (32) | 0.01 |
| Non-CR/non-PD | 27 (10) | 24 (9) | 0.77 |
| Progression disease | 44 (16) | 63 (23) | 0.05 |
| Not assessment | 11 (4) | 14 (5) | 0.68 |
| Objective response rate | 134 (49) | 86 (31) | < 0.001 |
| Disease control rate | 220 (80) | 198 (72) | 0.04 |

Abbreviations: ECOG, Eastern Cooperative Oncology Group; ICI, Immune checkpoint inhibitor; CR, Complete response; PD, Progression disease
