# Supplementary material for: Chemoimmunotherapy Outcomes and Prognostic Factors in Patients with Advanced, Low PD-L1–Expressing Non–Small Cell Lung Cancer
Source: Cancer Res Commun. 2025 Jul 23;5(7):1203–14. doi: 10.1158/2767-9764.CRC-25-0157 (PMC12284348; doi:10.1158/2767-9764.CRC-25-0157)
Supplement: Supplementary Table S5 — Univariate and multivariate analysis of Overall Survival and Progression-Free Survival in patients treated with Chemotherapy [file crc-25-0157_supplementary_table_s5_suppst5.docx]

**Supplementary Table S5. Univariate and multivariate analysis of Overall Survival and Progression-Free Survival in patients treated with Chemotherapy**

1. **Overall Survival**

| **Characteristics** | **Patients, No. (%)**  **(N = 275)** | **Median OS**  **(95% CI) months** | **Univariate analysis** | | **Multivariate analysis** | |
| --- | --- | --- | --- | --- | --- | --- |
|  |  |  | **HR (95% CI)** | ***P* Value** | **HR (95% CI)** | ***P* Value** |
| Age |  |  |  |  |  |  |
| < 75 | 211 | 16.7 (14.6, 20.3) | 0.81 (0.58-1.11) | 0.19 | 0.92 (0.63-1.33) | 0.65 |
| >= 75 | 64 | 17.2 (9.5, 20.8) | 1 [Reference] | NA | 1 [Reference] | NA |
| Sex |  |  |  |  |  |  |
| Female | 78 | 17.7 (15.3, 21.3) | 0.98 (0.73-1.33) | 0.92 | 1.07 (0.73-1.56) | 0.72 |
| Male | 197 | 16.1 (12.8, 18.8) | 1 [Reference] | NA | 1 [Reference] | NA |
| ECOG performance status |  |  |  |  |  |  |
| 0–1 | 256 | 17.0 (14.7, 19.3) | 0.73 (0.43-1.24) | 0.24 | 0.88 (0.49-1.59) | 0.67 |
| 2–4 | 19 | 15.9 (3.7, 28.8) | 1 [Reference] | NA | 1 [Reference] | NA |
| Smoking history |  |  |  |  |  |  |
| Never | 49 | 20.3 (15.4, 27.3) | 0.82 (0.57-1.19) | 0.3 | 0.93 (0.59-1.47) | 0.76 |
| Former/current | 226 | 16.0 (13.4, 18.1) | 1 [Reference] | NA | 1 [Reference] | NA |
| Histology |  |  |  |  |  |  |
| Squamous | 74 | 12.2 (8.4, 17.6) | 1.48 (1.10-1.99) | 0.01 | 1.49 (0.99-2.23) | 0.05 |
| Non-Squamous | 201 | 17.9 (15.9, 22.4) | 1 [Reference] | NA | 1 [Reference] | NA |
| EGFR mutation status |  |  |  |  |  |  |
| Positive | 51 | 17.7 (12.8, 22.7) | 1.00 (0.69-1.45) | 1 | 1.25 (0.82-1.89) | 0.3 |
| Negative | 187 | 16.7 (14.5, 20.3) | 1 [Reference] | NA | 1 [Reference] | NA |
| Brain metastases |  |  |  |  |  |  |
| Yes | 53 | 12.7 (9.4, 17.0) | 1.33 (0.94-1.88) | 0.11 | 1.37 (0.86-2.19) | 0.18 |
| No | 222 | 17.6 (15.4, 21.1) | 1 [Reference] | NA | 1 [Reference] | NA |
| Liver metastases |  |  |  |  |  |  |
| Yes | 35 | 10.0 (6.5, 16.0) | 1.65 (1.11-2.47) | 0.01 | 1.61 (1.01-2.58) | 0.045 |
| No | 240 | 17.6 (15.4, 20.8) | 1 [Reference] | NA | 1 [Reference] | NA |
| Prior radiation therapy |  |  |  |  |  |  |
| Yes | 58 | 17.2 (12.0, 22.6) | 1.01 (0.72-1.42) | 0.94 | 0.85 (0.52-1.37) | 0.49 |
| No | 217 | 16.7 (14.6, 19.5) | 1 [Reference] | NA | 1 [Reference] | NA |
| Proton pump inhibitor |  |  |  |  |  |  |
| Administered | 105 | 16.3 (12.7, 18.5) | 1.10 (0.83-1.45) | 0.51 | 1.12 (0.82-1.53) | 0.46 |
| Not administered | 170 | 17.2 (14.5, 21.3) | 1 [Reference] | NA | 1 [Reference] | NA |
| Antibiotics |  |  |  |  |  |  |
| Administered | 34 | 14.1 (8.1, 18.1) | 1.87 (1.27-2.74) | 0.001 | 2.06 (1.29-3.31) | 0.003 |
| Not administered | 241 | 17.2 (14.9, 21.3) | 1 [Reference] | NA | 1 [Reference] | NA |
| Steroids and/or immunosuppressant |  |  |  |  |  |  |
| Administered | 27 | 16.6 (6.4, 22.6) | 1.22 (0.76-1.95) | 0.42 | 1.02 (0.56-1.87) | 0.94 |
| Not administered | 248 | 17.0 (14.8, 19.5) | 1 [Reference] | NA | 1 [Reference] | NA |

Abbreviations: ICI, Immune checkpoint inhibitor; OS, overall survival; ECOG, Eastern Cooperative Oncology Group; EGFR, Epidermal growth factor receptor

1. **Progression-Free Survival**

| **Characteristics** | **Patients, No. (%)**  **(N = 275)** | **Median PFS**  **(95% CI) months** | **Univariate analysis** | | **Multivariate analysis** | |
| --- | --- | --- | --- | --- | --- | --- |
|  |  |  | **HR (95% CI)** | ***P* Value** | **HR (95% CI)** | ***P* Value** |
| Age |  |  |  |  |  |  |
| < 75 | 211 | 5.4 (4.4, 5.9) | 1.00 (0.74-1.35) | 0.99 | 0.99 (0.71-1.39) | 0.95 |
| >= 75 | 64 | 5.9 (4.3, 6.5) | 1 [Reference] | NA | 1 [Reference] | NA |
| Sex |  |  |  |  |  |  |
| Female | 78 | 5.6 (4.2, 6.3) | 0.94 (0.71-1.25) | 0.67 | 0.86 (0.61-1.23) | 0.41 |
| Male | 197 | 5.4 (4.4, 6.1) | 1 [Reference] | NA | 1 [Reference] | NA |
| ECOG performance status |  |  |  |  |  |  |
| 0–1 | 256 | 5.6 (4.7, 6.1) | 0.53 (0.32-0.89) | 0.01 | 0.71 (0.39-1.29) | 0.26 |
| 2–4 | 19 | 3.5 (1.8, 6.1) | 1 [Reference] | NA | 1 [Reference] | NA |
| Smoking history |  |  |  |  |  |  |
| Never | 49 | 6.1 (5.0, 9.7) | 0.78 (0.56-1.09) | 0.15 | 0.75 (0.50-1.13) | 0.17 |
| Former/current | 226 | 5.3 (4.3, 5.9) | 1 [Reference] | NA | 1 [Reference] | NA |
| Histology |  |  |  |  |  |  |
| Squamous | 74 | 5.0 (3.5, 5.9) | 1.14 (0.84-1.54) | 0.41 | 1.06 (0.69-1.62) | 0.79 |
| Non-Squamous | 201 | 5.6 (4.6, 6.3) | 1 [Reference] | NA | 1 [Reference] | NA |
| EGFR mutation status |  |  |  |  |  |  |
| Positive | 51 | 5.3 (3.7, 6.1) | 1.28 (0.92-1.77) | 0.15 | 1.54 (1.06-2.25) | 0.03 |
| Negative | 187 | 5.6 (4.5, 6.4) | 1 [Reference] | NA | 1 [Reference] | NA |
| Brain metastases |  |  |  |  |  |  |
| Yes | 53 | 3.7 (3.0, 6.9) | 1.17 (0.84-1.63) | 0.36 | 1.12 (0.73-1.72) | 0.59 |
| No | 222 | 5.6 (4.7, 6.1) | 1 [Reference] | NA | 1 [Reference] | NA |
| Liver metastases |  |  |  |  |  |  |
| Yes | 35 | 2.6 (1.5, 4.0) | 2.47 (1.70-3.60) | < 0.001 | 2.48 (1.63-3.76) | < 0.001 |
| No | 240 | 5.8 (5.0, 6.3) | 1 [Reference] | NA | 1 [Reference] | NA |
| Prior radiation therapy |  |  |  |  |  |  |
| Yes | 58 | 5.0 (3.5, 8.0) | 0.94 (0.68-1.31) | 0.73 | 0.93 (0.59-1.47) | 0.76 |
| No | 217 | 5.6 (4.6, 6.1) | 1 [Reference] | NA | 1 [Reference] | NA |
| Proton pump inhibitor |  |  |  |  |  |  |
| Administered | 105 | 5.9 (4.4, 6.5) | 0.93 (0.71-1.21) | 0.58 | 1.02 (0.75-1.39) | 0.89 |
| Not administered | 170 | 5.4 (4.3, 5.9) | 1 [Reference] | NA | 1 [Reference] | NA |
| Antibiotics |  |  |  |  |  |  |
| Administered | 34 | 4.6 (3.5, 5.4) | 1.57 (1.06-2.33) | 0.02 | 1.64 (1.01-2.67) | 0.04 |
| Not administered | 241 | 5.6 (4.6, 6.3) | 1 [Reference] | NA | 1 [Reference] | NA |
| Steroids and/or immunosuppressant |  |  |  |  |  |  |
| Administered | 27 | 4.0 (1.9, 6.9) | 1.26 (0.81-1.95) | 0.31 | 1.07 (0.63-1.82) | 0.8 |
| Not administered | 248 | 5.6 (4.7, 6.1) | 1 [Reference] | NA | 1 [Reference] | NA |

Abbreviations: ICI, Immune checkpoint inhibitor; PFS, progression-free survival; ECOG, Eastern Cooperative Oncology Group; EGFR, Epidermal growth factor receptor
