# Supplementary material for: Chemoimmunotherapy Outcomes and Prognostic Factors in Patients with Advanced, Low PD-L1–Expressing Non–Small Cell Lung Cancer
Source: Cancer Res Commun. 2025 Jul 23;5(7):1203–14. doi: 10.1158/2767-9764.CRC-25-0157 (PMC12284348; doi:10.1158/2767-9764.CRC-25-0157)
Supplement: Supplementary Table S6 — Characteristics in the Liver and non-Liver metastases Groups adjusted by propensity score matching [file crc-25-0157_supplementary_table_s6_suppst6.docx]

**Supplementary Table S6. Characteristics in the Liver and non-Liver metastases Groups adjusted by propensity score matching**

| **Characteristics** | **ICI plus Chemotherapy**  **N = 38**  **No. (%)** | **Chemotherapy**  **N = 38**  **No. (%)** | ***P* Value** |
| --- | --- | --- | --- |
| Median age (range) | 69 [36-87] | 66 [39-82] | 0.52 |
| Sex |  |  |  |
| Female | 10 (26) | 10 (26) | 1.0 |
| Male | 28 (74) | 28 (74) |  |
| ECOG performance status |  |  |  |
| 0–1 | 35 (92) | 35 (92) | 1.0 |
| 2–4 | 3 (8) | 3 (8) |  |
| Smoking history |  |  |  |
| Never | 8 (21) | 4 (11) | 0.35 |
| Former/current | 30 (79) | 34 (90) |  |
| Histology |  |  |  |
| Squamous | 11 (29) | 11 (29) | 1.0 |
| Non-Squamous | 27 (71) | 27 (71) |  |
| Disease stage |  |  |  |
| IIIB– IV | 36 (95) | 31 (82) | 0.15 |
| Recurrence | 2 (5) | 7 (18) |  |
| EGFR mutation status |  |  |  |
| Positive | 9 (24) | 6 (16) | 0.66 |
| Negative | 25 (66) | 29 (76) |  |
| Unknown | 4 (11) | 3 (8) |  |
| Brain metastases |  |  |  |
| Yes | 14 (37) | 9 (24) | 0.32 |
| No | 24 (63) | 29 (76) |  |
| Proton pump inhibitor |  |  |  |
| Administered | 14 (37) | 13 (34) | 1.0 |
| Not administered | 24 (63) | 25 (66) |  |
| Steroids and/or immunosuppressant |  |  |  |
| Administered | 1 (3) | 2 (5) | 1.0 |
| Not administered | 37 (97) | 36 (95) |  |

1. **The Liver metastases Group**

Abbreviations: ICI, Immune checkpoint inhibitor; ECOG, Eastern Cooperative Oncology Group; EGFR, Epidermal growth factor receptor

1. **The non-Liver metastases Group**

| **Characteristics** | **ICI plus Chemotherapy**  **N = 234**  **No. (%)** | **Chemotherapy**  **N = 234**  **No. (%)** | ***P* Value** |
| --- | --- | --- | --- |
| Median age (range) | 71 [36-89] | 69 [39-86] | 0.3 |
| Sex |  |  |  |
| Female | 69 (30) | 80 (34) | 0.32 |
| Male | 165 (71) | 154 (66) |  |
| ECOG performance status |  |  |  |
| 0–1 | 216 (92) | 216 (92) | 1.0 |
| 2–4 | 18 (8) | 18 (8) |  |
| Smoking history |  |  |  |
| Never | 53 (23) | 51 (22) | 0.91 |
| Former/current | 181 (77) | 183 (78) |  |
| Histology |  |  |  |
| Squamous | 50 (21) | 53 (23) | 0.82 |
| Non-Squamous | 184 (79) | 181 (77) |  |
| Disease stage |  |  |  |
| IIIB– IV | 184 (79) | 185 (79) | 1.0 |
| Recurrence | 50 (21) | 49 (21) |  |
| EGFR mutation status |  |  |  |
| Positive | 38 (16) | 38 (16) | 1.0 |
| Negative | 196 (84) | 196 (84) |  |
| Brain metastases |  |  |  |
| Yes | 39 (17) | 40 (17) | 1.0 |
| No | 195 (83) | 194 (83) |  |
| Proton pump inhibitor |  |  |  |
| Administered | 89 (38) | 90 (39) | 1.0 |
| Not administered | 145 (62) | 144 (62) |  |
| Steroids and/or immunosuppressant |  |  |  |
| Administered | 24 (10) | 22 (9) | 0.88 |
| Not administered | 210 (90) | 212 (91) |  |

Abbreviations: ICI, Immune checkpoint inhibitor; ECOG, Eastern Cooperative Oncology Group
