# Supplementary material for: Chemoimmunotherapy Outcomes and Prognostic Factors in Patients with Advanced, Low PD-L1–Expressing Non–Small Cell Lung Cancer
Source: Cancer Res Commun. 2025 Jul 23;5(7):1203–14. doi: 10.1158/2767-9764.CRC-25-0157 (PMC12284348; doi:10.1158/2767-9764.CRC-25-0157)
Supplement: Supplementary Table S7 — Characteristics in the Antibiotics and non-Antibiotics Groups adjusted by propensity score matching [file crc-25-0157_supplementary_table_s7_suppst7.docx]

**Supplementary Table S7. Characteristics in the Antibiotics and non-Antibiotics Groups adjusted by propensity score matching**

| **Characteristics** | **ICI plus Chemotherapy**  **N = 37**  **No. (%)** | **Chemotherapy**  **N = 37**  **No. (%)** | ***P* Value** |
| --- | --- | --- | --- |
| Median age (range) | 72 [49-89] | 72 [48-83] | 0.77 |
| Sex |  |  |  |
| Female | 7 (19) | 6 (16) | 1.0 |
| Male | 30 (81) | 31 (84) |  |
| ECOG performance status |  |  |  |
| 0–1 | 30 (81) | 31 (84) | 1.0 |
| 2–4 | 7 (19) | 6 (16) |  |
| Smoking history |  |  |  |
| Never | 5 (14) | 3 (8) | 0.71 |
| Former/current | 32 (86) | 34 (92) |  |
| Histology |  |  |  |
| Squamous | 13 (35) | 17 (46) | 0.48 |
| Non-Squamous | 24 (65) | 20 (54) |  |
| Disease stage |  |  |  |
| IIIB– IV | 36 (97) | 34 (92) | 0.62 |
| Recurrence | 1 (3) | 3 (8) |  |
| EGFR mutation status |  |  |  |
| Positive | 0 (0) | 1 (3) | 0.57 |
| Negative | 30 (81) | 28 (76) |  |
| Unknown | 7 (19) | 8 (22) |  |
| Brain metastases |  |  |  |
| Yes | 9 (24) | 5 (14) | 0.37 |
| No | 28 (76) | 32 (86) |  |
| Proton pump inhibitor |  |  |  |
| Administered | 17 (46) | 13 (35) | 0.48 |
| Not administered | 20 (54) | 24 (65) |  |
| Steroids and/or immunosuppressant |  |  |  |
| Administered | 4 (11) | 11 (30) | 0.08 |
| Not administered | 33 (89) | 26 (70) |  |

1. **The Antibiotics Group**

Abbreviations: ICI, Immune checkpoint inhibitor; ECOG, Eastern Cooperative Oncology Group; EGFR, Epidermal growth factor receptor

1. **The non-Antibiotics Group**

| **Characteristics** | **ICI plus Chemotherapy**  **N = 236**  **No. (%)** | **Chemotherapy**  **N = 236**  **No. (%)** | ***P* Value** |
| --- | --- | --- | --- |
| Median age (range) | 69 [36-85] | 69 [39-88] | 0.28 |
| Sex |  |  |  |
| Female | 66 (28) | 70 (30) | 0.76 |
| Male | 170 (72) | 166 (70) |  |
| ECOG performance status |  |  |  |
| 0–1 | 221 (94) | 221 (94) | 1.0 |
| 2–4 | 15 (6) | 15 (6) |  |
| Smoking history |  |  |  |
| Never | 43 (18) | 44 (19) | 1.0 |
| Former/current | 193 (82) | 192 (81) |  |
| Histology |  |  |  |
| Squamous | 58 (33) | 62 (26) | 0.75 |
| Non-Squamous | 178 (75) | 174 (74) |  |
| Disease stage |  |  |  |
| IIIB– IV | 183 (78) | 186 (79) | 0.82 |
| Recurrence | 53 (22) | 50 (21) |  |
| EGFR mutation status |  |  |  |
| Positive | 39 (17) | 41 (17) | 0.97 |
| Negative | 168 (71) | 166 (70) |  |
| Unknown | 29 (12) | 29 (12) |  |
| Brain metastases |  |  |  |
| Yes | 50 (21) | 47 (20) | 0.82 |
| No | 186 (79) | 189 (80) |  |
| Proton pump inhibitor |  |  |  |
| Administered | 78 (33) | 88 (37) | 0.39 |
| Not administered | 158 (67) | 148 (63) |  |
| Steroids and/or immunosuppressant |  |  |  |
| Administered | 17 (7) | 18 (8) | 1.0 |
| Not administered | 219 (93) | 218 (92) |  |

Abbreviations: ICI, Immune checkpoint inhibitor; ECOG, Eastern Cooperative Oncology Group
