# Supplementary material for: Chemoimmunotherapy Outcomes and Prognostic Factors in Patients with Advanced, Low PD-L1–Expressing Non–Small Cell Lung Cancer
Source: Cancer Res Commun. 2025 Jul 23;5(7):1203–14. doi: 10.1158/2767-9764.CRC-25-0157 (PMC12284348; doi:10.1158/2767-9764.CRC-25-0157)
Supplement: Supplementary Table S8 — Details of the antimicrobial administration history [file crc-25-0157_supplementary_table_s8_suppst8.docx]

**Supplementary Table S8. Details of the antimicrobial administration history**

|  | **Patients, No. (%)**  **(N = 126)** |
| --- | --- |
|  |  |
| Types of Antibiotics |  |
| Beta-lactam | 86 (68) |
| Quinolone | 18 (14) |
| Macrolide | 10 (8) |
| Trimethoprim-sulfamethoxazole | 4 (3) |
| Lincosamide | 3 (2) |
| Tetracycline | 2 (2) |
| Glycopeptide | 1 (0.8) |
| Unknown | 2 (2) |
| Reason for Antibiotic Use |  |
| Pneumonitis | 61 (48) |
| Prophylactic medications associated with biopsy | 40 (32) |
| Unknown source of infection | 6 (5) |
| Prevention of Pneumocystis pneumonia | 4 (3) |
| Skin rash treatment | 3 (2) |
| Colitis | 1 (0.8) |
| Sinusitis | 1 (0.8) |
| Unknown | 10 (8) |
| Main treatment modalities |  |
| Hospitalization | 71 (56) |
| Outpatient visits | 39 (31) |
| Unknown | 16 (13) |
| Route of dosing |  |
| Oral medicine | 59 (47) |
| Intravenous administration | 65 (52) |
| Unknown | 2 (2) |
| Median duration of antibiotics therapy (days, range) | 7 [1-584] |
| Patients receiving concomitant probiotics while on ICI | 6 (5) |

Abbreviations: ICI, Immune checkpoint inhibitor
