# Supplementary material for: Chemoimmunotherapy Outcomes and Prognostic Factors in Patients with Advanced, Low PD-L1–Expressing Non–Small Cell Lung Cancer
Source: Cancer Res Commun. 2025 Jul 23;5(7):1203–14. doi: 10.1158/2767-9764.CRC-25-0157 (PMC12284348; doi:10.1158/2767-9764.CRC-25-0157)
Supplement: Supplementary Table S9 — Incidence of interstitial pneumonitis as an adverse event in the population adjusted by propensity score matching [file crc-25-0157_supplementary_table_s9_suppst9.docx]

**Supplementary Table S9. Incidence of interstitial pneumonitis as an adverse event in the population adjusted by propensity score matching**

|  | **All patients, N = 550** | | **ATB group, N = 74** | |
| --- | --- | --- | --- | --- |
|  | **ICI plus**  **Chemotherapy**  **N = 275**  **No. (%)** | **Chemotherapy**  **N = 275**  **No. (%)** | **ICI plus**  **Chemotherapy**  **N = 37**  **No. (%)** | **Chemotherapy**  **N = 37**  **No. (%)** |
| All | 43 (16) | 26 (9) | 8 (22) | 3 (8) |
| Grade 1 | 6 (2) | 8 (3) | 0 (0) | 0 (0) |
| Grade 2 | 20 (7) | 3 (1) | 2 (5) | 2 (5) |
| Grade 3 | 14 (5) | 14 (5) | 3 (8) | 1 (3) |
| Grade 4 | 3 (1) | 0 (0) | 1 (3) | 0 (0) |
| Grade 5 | 0 (0) | 1 (0.3) | 2 (5) | 0 (0) |

Abbreviations: ICI, Immune checkpoint inhibitor; ATB, antibiotics
