# Supplementary material for: Chemoimmunotherapy Outcomes and Prognostic Factors in Patients with Advanced, Low PD-L1–Expressing Non–Small Cell Lung Cancer
Source: Cancer Res Commun. 2025 Jul 23;5(7):1203–14. doi: 10.1158/2767-9764.CRC-25-0157 (PMC12284348; doi:10.1158/2767-9764.CRC-25-0157)
Supplement: Supplementary Table S10 — Discontinuation due to adverse events and ECOG Performance status deterioration due to treatment in the population adjusted by propensity score matching [file crc-25-0157_supplementary_table_s10_suppst10.docx]

**Supplementary Table S10. Discontinuation due to adverse events and ECOG Performance status deterioration due to treatment in the population adjusted by propensity score matching**

|  | **ICI plus**  **Chemotherapy**  **No. (%)** | **Chemotherapy**  **No. (%)** | ***P* Value** |
| --- | --- | --- | --- |
| Discontinuation due to adverse events |  |  |  |
| All patients, N = 550 | 72 (26) | 38 (14) | < 0.001 |
| LM group, N = 76 | 7 (18) | 3 (8) | 0.31 |
| ATB group, N = 74 | 9 (24) | 4 (11) | 0.22 |
| ECOG Performance status deterioration due to treatment |  |  |  |
| All patients, N = 550 | 8 (3) | 7 (3) | 1.0 |
| LM group, N = 76 | 1 (3) | 1 (3) | 1.0 |
| ATB group, N = 74 | 2 (5) | 2 (5) | 1.0 |

Abbreviations: ICI, Immune checkpoint inhibitor; ATB, antibiotics; ECOG, Eastern Cooperative Oncology Group; LM, liver metastases
