# Supplementary material for: Chemoimmunotherapy Outcomes and Prognostic Factors in Patients with Advanced, Low PD-L1–Expressing Non–Small Cell Lung Cancer
Source: Cancer Res Commun. 2025 Jul 23;5(7):1203–14. doi: 10.1158/2767-9764.CRC-25-0157 (PMC12284348; doi:10.1158/2767-9764.CRC-25-0157)
Supplement: Supplementary Figure S1 — Study Flow Diagram [file crc-25-0157_supplementary_figure_s1_suppsf1.docx]

**Supplementary Figure S1. Study Flow Diagram**

ICI, immune checkpoint inhibitor; PD-L1, programmed cell death ligand; EGFR, epidermal growth factor receptor; TKI, tyrosine kinase inhibitor
