# Supplementary material for: Chemoimmunotherapy Outcomes and Prognostic Factors in Patients with Advanced, Low PD-L1–Expressing Non–Small Cell Lung Cancer
Source: Cancer Res Commun. 2025 Jul 23;5(7):1203–14. doi: 10.1158/2767-9764.CRC-25-0157 (PMC12284348; doi:10.1158/2767-9764.CRC-25-0157)
Supplement: Supplementary Figure S2 — Overall survival and Progression-free survival of 851 patients according to treatments [file crc-25-0157_supplementary_figure_s2_suppsf2.docx]

**Supplementary Figure S2. Overall survival and Progression-free survival of 851 patients according to treatments**

eFigure 2. The Kaplan-Meier curves provide estimates of OS (A) and PFS (B) of the 851 patients, stratified by treatment with ICI plus chemotherapy and chemotherapy.

ICI, immune checkpoint inhibitor; Chemo, chemotherapy
