# Supplementary material for: Chemoimmunotherapy Outcomes and Prognostic Factors in Patients with Advanced, Low PD-L1–Expressing Non–Small Cell Lung Cancer
Source: Cancer Res Commun. 2025 Jul 23;5(7):1203–14. doi: 10.1158/2767-9764.CRC-25-0157 (PMC12284348; doi:10.1158/2767-9764.CRC-25-0157)
Supplement: Supplementary Figure S3 — Incidence of Interstitial Pneumonitis as an adverse event [file crc-25-0157_supplementary_figure_s3_suppsf3.docx]

**Supplementary Figure S3. Incidence of Interstitial Pneumonitis as an adverse event**

eFigure 3. Incidence of interstitial pneumonitis in all patients treated with ICI plus chemotherapy (A) and chemotherapy (B) and in patients with a history of antibiotics (ATB) use treated with ICI plus chemotherapy (C) and chemotherapy (D).
